# To Use or Not to Use a COVID-19 Contact Tracing App: Mixed Methods Survey in Wales

**DOI:** 10.2196/29181

**Published:** 2021-11-22

**Authors:** Kerina Jones, Rachel Thompson

**Affiliations:** 1 Swansea University Swansea United Kingdom

**Keywords:** COVID-19, survey, Wales, contact tracing, app, mHealth, mobile apps, digital health, public health

## Abstract

**Background:**

Many countries remain in the grip of the COVID-19 global pandemic, with a considerable journey still ahead toward normalcy and free mobility. Contact tracing smartphone apps are among a raft of measures introduced to reduce spread of the virus, but their uptake depends on public choice.

**Objective:**

The objective of this study was to ascertain the views of citizens in Wales on their intended use of a COVID-19 contact tracing smartphone app, including self-proposed reasons for or against use and what could lead to a change of decision.

**Methods:**

We distributed an anonymous survey among 4000 HealthWise Wales participants in May 2020. We adopted a mixed methods approach: responses to closed questions were analyzed using descriptive and inferential statistics; open question responses were analyzed and grouped into categories.

**Results:**

A total of 976 (24.4%) people completed the survey. Smartphone usage was 91.5% overall, but this varied among age groups. In total, 97.1% were aware of contact tracing apps, but only 67.2% felt sufficiently informed. Furthermore, 55.7% intended to use an app, 23.3% refused, and 21.0% were unsure. The top reasons for app use were as follows: controlling the spread of the virus, mitigating risks for others and for oneself, and increasing freedoms. The top reasons against app use were as follows: mistrusting the government, concerns about data security and privacy, and doubts about efficacy. The top response for changing one’s mind about app use from being willing to being unwilling was that nothing would; that is, they felt that nothing would cause them to become unwilling to use a contact tracing app. This was also the top response for changing one’s mind from being unwilling to being willing to use contact tracing apps. Among those who were unsure of using contact tracing apps, the top response was the need for more information.

**Conclusions:**

Respondents demonstrated a keenness to help themselves, others, society, and the government to avoid contracting the virus and to control its spread. However, digital inclusion varied among age groups, precluding participation for some people. Nonetheless, unwillingness was significant, and considering the nature of the concerns raised and the perceived lack of information, policy and decision-makers need to do more to act openly, increase communication, and demonstrate trustworthiness if members of the public are to be confident in using an app.

## Introduction

In common with many countries worldwide, Wales remains in the grip of the COVID-19 global pandemic, with a considerable journey still ahead toward normalcy and free mobility. With a population of 3.1 million individuals, Wales accounts a relatively small fraction of the 66.8 million individuals in the United Kingdom [1]. Nonetheless, at the time of writing, Wales has had a cumulative total of over 200,000 cases and over 5400 deaths due to COVID-19 with a confirmed positive test [2]. The Senedd—the Welsh Government—is committed to the use of contact tracing smartphone app technology as part of a raft of measures to control and reduce spread of the virus. Along with the direct measures of individual testing and vaccinations, these also include society-wide travel restrictions; closure of schools, nonessential shops, and businesses; a track-and-trace system; and working from home wherever possible, such that the entire country has been on the highest level of lockdown used by the Senedd (alert level 4) [3]. As such, this is a novel situation in general and specifically concerning the introduction of a contact tracing app.

There has been considerable debate in the media about the use of contact tracing smartphone apps during the pandemic. Some concerns that have been raised are the perceived risk to individual privacy, data security, and the ethics of automated data collection with a person’s own private device [4]. Although contact tracing is a longstanding part of public health surveillance for infectious diseases, the standard methods are seen as too slow in a pandemic situation when used alone. The use of smartphone apps is proposed to capture information more quickly and expedite rapid information sharing to enable action to prevent further spread [4]. In the spirit of openness and because of the concerns raised, there is considerable interest in citizens’ views on the use of these smartphone apps. Since majority uptake is needed to achieve maximum effectiveness, it is important to gauge and understand citizens’ views on the acceptability of contact tracing apps for smartphones.

The published literature shows that there have been large-scale quantitative studies with the public and small-scale qualitative studies to gain insight into citizens’ perceptions about the use of an app in various countries. These have included a variety of study designs: surveys providing a menu of options for respondents to indicate their reasons for or against the use of an app [5-7]; discrete choice experiments where respondents are asked to select options based on trade-offs [8]; and focus groups where individuals were able to present their own reasons for or against an app [9]. The focus groups study conducted by Williams et al [9] included participants from all 4 UK countries but naturally had a small sample size. The multi-country survey carried out by Altmann et al [5] included people from the United Kingdom but did not specify the breakdown by country. Since public opinion on self-determination, personal choice, and responsibility can vary by culture and government administration across countries, we wanted to address the knowledge gap by finding out more about Welsh citizens’ views. Our research question was as follows: What are the views and intentions of members of the Welsh public in relation to using a contact tracing app? To address this question, we designed a mixed methods study that included a survey composed of a combination of closed and open questions. In particular, to generate rich information, we allowed the participants to provide open responses for or against the use of an app and on what would lead them to change their mind regarding app use.

The objective of this study was to ascertain the views of citizens in Wales on their intended use of a COVID‑19 contact tracing smartphone app, including self-proposed reasons for or against it, and what could lead to a change of decision.

## Methods

We designed and distributed an anonymous survey among 4000 HealthWise Wales (HWW) participants. HWW is a cohort of approximately 40,000 people who have signed up to help shape the health and well-being of future generations in Wales. Compared with the wider population, there was a higher percentage of HWW participants older than 45 years. The percentage of women was higher than that in the general population (72% vs 51%). The percentage of participants in ethnic groups other than White ethnicity (2%) matched that in the Welsh population. Around half of participants are in higher managerial or professional occupations, which is significantly greater than the general population; however, each quintile of the Welsh Index of Multiple Deprivation was represented [10,11]. The survey was released on the internet on the TypeForm site and piloted to check for consistency before being released. To yield a rapid response, we asked HWW to select a random sample from their full cohort, correctively weighted for responses among men, and ethnic minorities of all genders as these groups were known to be underrepresented in the HWW cohort. Ethical approval was not required for the study, as no identifiable information was sought.

The survey was released from May 22 to 28, 2020, and was closed when the response rate had tailed off. At this time, the United Kingdom was testing the NHSX app which was intended to operate on a centralized data collection model. This model was abandoned shortly after the survey was conducted, and the National Health Service (NHS) COVID-19 contact tracing app (operating on a decentralized model) was rolled out in England and Wales on September 24, 2020. Both models collect data via Bluetooth technology, but in a decentralized model, data processing is performed on the smartphone rather than being transferred to and stored in a central database.

All the survey questions were in a closed or structured format apart from those about reasons for being willing or unwilling to use a contact tracing app, and reasons for a change of mind regarding app use. Participants were invited to provide up to 3 reasons for or against app use and were not asked to rank them. They were asked for one reason for why they might change their mind. These responses were open in the free-text format and were analyzed and grouped manually by a consensus with 2 researchers. The quantitative data were analyzed using SPSS (version 26), using descriptive (n [%]) and inferential (chi-square) statistics. A list of survey questions is provided in Multimedia Appendix 1.

## Results

### Basic Demographics

**Figure 1:**
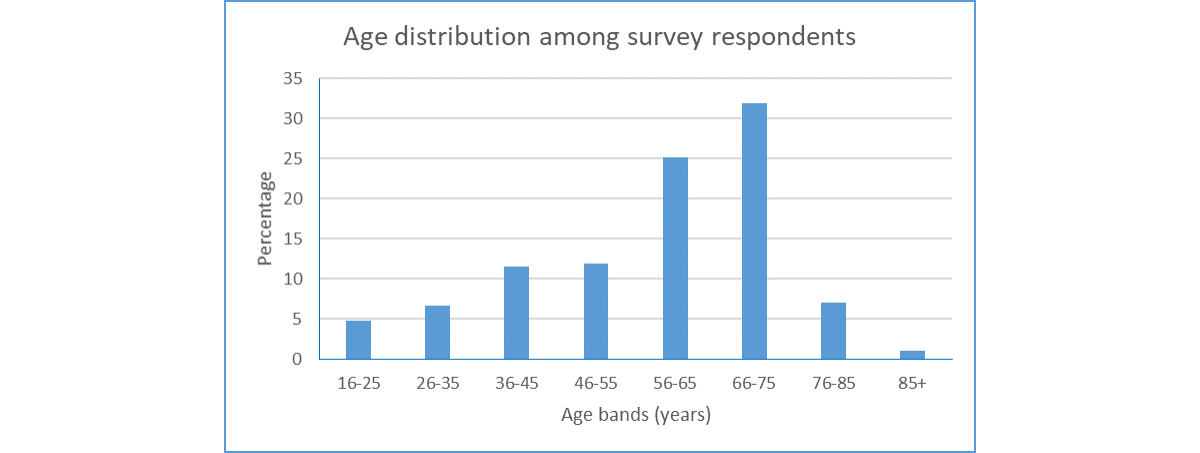
Age distribution among the survey respondents. In total, 974 people (of 976 in total) provided their ages in 10-year bands.

A total of 976 (24.4%) full survey responses were received from across all main postcode areas of Wales (Cardiff, Llandrindod, Wrexham, Newport, Swansea, and Shrewsbury). Only the first part of the postcode was requested to preserve anonymity. Among 968 respondents, 504 (52.1%) responded as being male, 461 (47.6%) as being female, and 3 (0.3%) as being nonbinary. In terms of ethnicity (N=965), 923 (95.5%) identified as White, 7 (0.7%) as being Asian or Asian British, 4 (0.4%) as being Black, African, Black British, or Caribbean, 26 (2.7%) as being mixed or of multiple ethnic groups, and 5 (0.5%) as being of other ethnicities. The age distribution of respondents (N=974) is shown in Figure 1.

### Smartphone Usage

Smartphone usage was reported as 91.5% overall, but this varied by age groups (*P*<.001). It was over 95% in age groups of up to 55 years, approximately 90% among individuals aged 56-75 years, 78% among the individuals aged 76-85 years, and only 40% among those aged over 85 years.

### COVID-19 Risk and Experience

**Figure 2:**
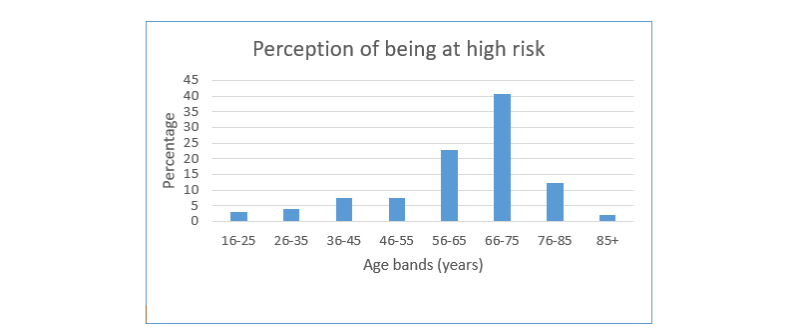
Respondents’ perceptions of being high risk of COVID-19 infection. In total, 971 respondents (of 976 in total) provided their perception of their personal risk of COVID-19.

Of 971 respondents, 291 (29.8%) considered themselves at high risk of infection and 680 (69.7%) did not believe that they were at high risk. There was no significant difference in the responses among all ethnic groups. Minority ethnicities were considered a single group in this instance as the numbers among each group were small. The distribution of these responses showing differences in the perception of personal risk among these age bands, with older people being at higher risk (*P*<.001), is shown in Figure 2. Among 971 respondents, 34 (3.5%) indicated that they had had COVID-19, and 35.7% indicated that they knew someone who had been infected. Both of these questions addressed whether COVID-19 was diagnosed or self-reported by these individuals.

### Knowledge and Use of Apps

Participants were asked about their knowledge and use of symptom tracking apps before proceeding to focus on contact tracing apps. A common symptom tracking app used in the United Kingdom is operated by the COVID Symptom Study, commonly referred to as “the Zoe app” [12]. In total, 974 (94%) respondents indicated that they were familiar with symptom tracking apps and 910 (37.7%) respondents used the app. Questions proceeded to ask about the awareness of plans for introducing a contact tracing app and whether people felt they had sufficient knowledge of potential benefits and risks. A total of 974 (97.1%) respondents were aware, and 973 (67.2%) respondents felt that they had sufficient knowledge. This was followed by a question about participants’ plans to use a contact tracing app if one were to be introduced. The number of responses to this question was only 652, of whom almost three-fourth (73.9%) indicated that they would do so, 12.7% refused, and 13.3% were unsure. Subsequent questions asked respondents about the reasons for their decision, and considering them, a yes/no/unsure response was inferred to the nonresponders from their reasons given for or against an app, yielding a total to 970 responses. The remaining 6 could not be inferred, as the respondents had not provided reasons. The inclusion of the inferred decisions resulted in the proportion for “yes” being reduced to just over half (55.7%), with the proportion for “no” increasing to 23.3% and that of “unsure” to 21.0% (Table 1).

There were no significant differences in the willingness to use a contact tracing app based on ethnicity or main postcode area. However, intentions varied by sex and by age. Females were more likely to be willing to use a contact tracing app than males (*P*=.045). Younger age groups tended to be less willing to use a contact tracing app than older age groups (*P*=.01). However, when assessed by sex, age was only a significant factor for females (*P*=.01).

**Table 1:** Respondents’ intentions on the use of a COVID-19 contact tracing app (N=970). This table shows the numbers and percentages of people intending to use, not intending to use, and unsure about using a COVID-19 contact tracing app.

Response type		Responses, n (%)
**Direct responses (n=652)**		
	Yes	482 (73.9)
	No	83 (12.7)
	Unsure	87 (13.3)
**Inferred from reasons (n=318)**		
	Yes	58 (18.2)
	No	143 (45.0)
	Unsure	117 (36.8)
**Total responses (N=970)**		
	Yes	540 (55.7)
	No	226 (23.3)
	Unsure	204 (21.0)

### Reasons for Being Willing to Use a Contact Tracing App

**Figure 3:**
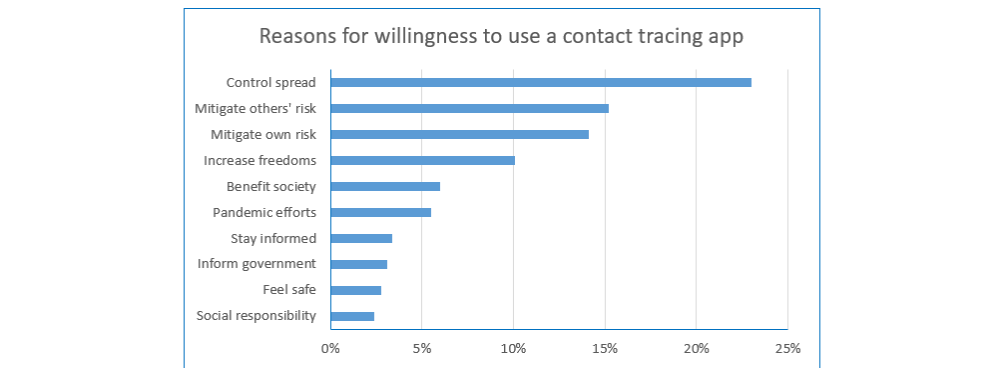
Respondents’ reasons for their willingness to use a COVID-19 contact tracing app. Participants provided their own, open responses on their reasons for being willing to use a contact tracing app. The top 10 reasons are shown here as percentages of those willing (N=540).

The top 10 reasons among people willing to use a contact tracing app are shown in Figure 3. Since the reasons were not requested to be ranked, we treated all reasons equally. As shown, the top reason was to control spread of the virus, followed by mitigating risk for others and for oneself, and a desire to increase freedoms.

### Quoted Responses From Willing Respondents

Some quoted responses from willing participants are given here as illustrations of their viewpoints.

#### Controlling Spread

I feel it will be an essential part of combating the spread of the virus, and gives me an element of control and decision making.

We need to emulate those countries that have managed to control their pandemic by use of this type of technology.

To help stop the spread of COVID-19 and to help inform decision to ease lockdowns.

#### Mitigating Risk

I wish to be able to move safely in my residential area; wish to know if I have been in contact with anyone diagnosed with the virus; wish to keep up to date with latest developments.

For my own peace of mind.

For my own safety and that of others & so that scientists have good data.

#### Increasing Freedoms

I would use one as I am keen to get the country going again.

Want lockdown to end, want pubs back open.

To help to overcome Covid, get back to work and enable a more normal life.

#### Other Reasons

We collectively owe it to our country to participate in track and trace to improve our chances of getting on top of Covid19.

Effective contact tracing, alongside widespread virus testing, is the best answer we have to managing the ongoing Covid 19 pandemic.

The sooner everyone takes responsibility for learning as much as possible about COVID 19 the sooner we’ll control it.

### Reasons for Being Unwilling to Use a Contact Tracing App

**Figure 4:**
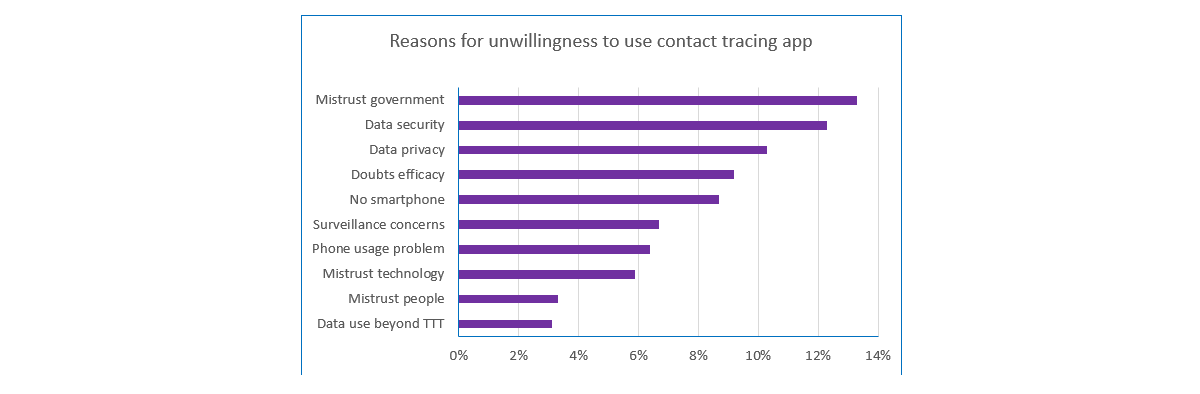
Respondents’ reasons for their unwillingness to use a COVID-19 contact tracing app. Participants provided their own, open responses on their reasons for being unwilling to use a contact tracing app. The top 10 reasons are shown here as percentages of those unwilling (N=226). TTT: test, track, and trace.

The top 10 reasons among those unwilling to use a contact tracing app are shown in Figure 4. Again, all reasons were treated equally. As shown, the top reason was mistrust in the government, followed by concerns about data security, data privacy, and app efficacy.

### Quoted Responses From Unwilling Respondents

Some quotations from unwilling participants are given here as illustrations of their viewpoints.

#### Mistrusting Government

I do not like the idea of the Government storing my data on a centralised system.

Creepy, 1984 stuff. Given how incompetent and chaotic Westminster's response to the virus has been so far, I wouldn't bet on information remaining confidential.

I have zero faith that the Westminster administration would not use the data for purposes other than tracing the virus.

#### Data Security

An app forcibly enabling Bluetooth which is inherently insecure is not something I will let happen on my phone.

Not confident my information won't be hacked or misused.

#### Data Privacy

Insufficient evidence of preservation of privacy and lack of adverse effects on device performance / battery life / security.

I worry about personal privacy.

#### Doubts About Efficacy

I am concerned that I may get into a cycle of being informed to self isolate multiple times because I might have been near somebody who may have the virus as I'm shopping for my wife and several neighbours once a week at a supermarket.

Info is confusing. Not willing to self isolate until person I've been in close contact with has confirmed COVID-19.

#### Other Reasons

I am not paying for a smartphone because of the stupid [expletive] in government won’t make an app that can be used on all mobile phones.

Concerns about data held centrally.

Mobile coverage in my area is patchy, this could be detrimental to the effectiveness of the app if you can't get a signal.

Risk of false warnings by malicious persons.

### Change of Decision

**Figure 5:**
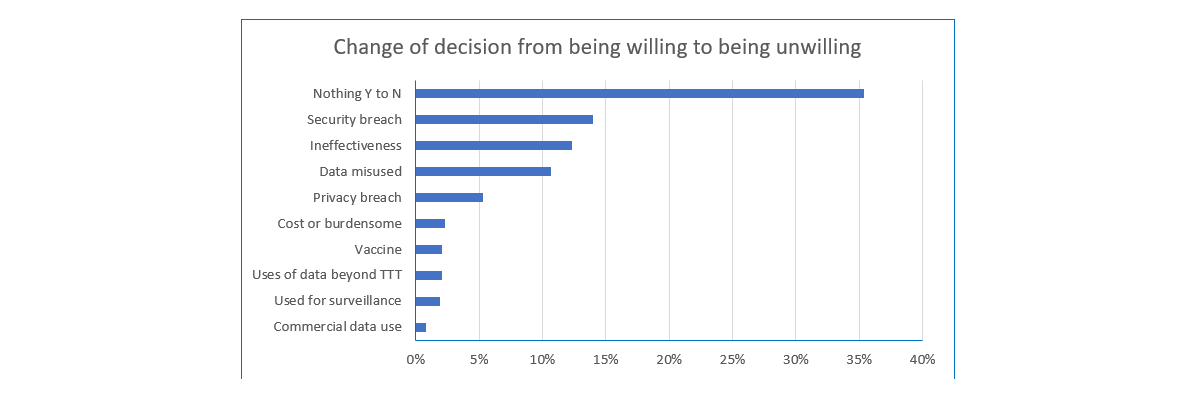
Participants’ responses to what would change their mind on app use from willing to unwilling. Participants who expressed willingness to use an app (N=540) provided their own, open responses on what would make them change to being unwilling to use a contact tracing app. The top 10 reasons are shown here. TTT: test, track, and trace.

When asked what would change their mind from being willing to being unwilling to use a contact tracing app, participants gave a range of reasons, with the top 10 reasons shown in Figure 5. As shown, the most frequent response to this question was nothing would; that is, they felt that nothing would cause them to become unwilling to use a contact tracing app. This was followed by a security breach, if the app proved ineffective, and if data were misused.

**Figure 6:**
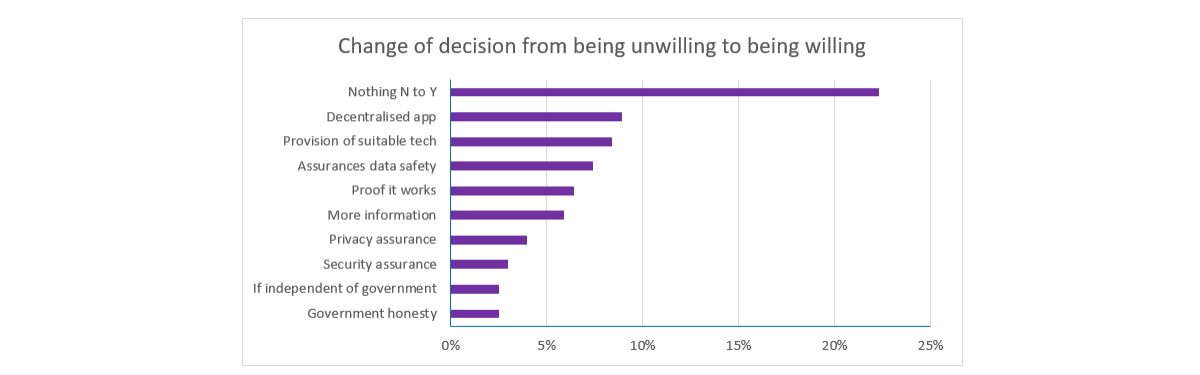
Participants’ responses to what would change their mind on app use from being unwilling to being willing. Participants who expressed unwillingness to use an app (N=226) provided their own open responses on what would make them change their mind to being willing to use a contact tracing app. The top 10 reasons are shown here.

When asked what would change their mind from being unwilling to being willing to use a contact tracing app, there were a variety of reasons, with the top 10 reasons shown in Figure 6. Again, the most frequent response to this question was that nothing would; that is, they felt that nothing would cause them to become willing to use a contact tracing app. This was followed by a preference for a decentralized app, the provision of suitable tech (ie, a smartphone, a network connection, or both), and assurances of data safety.

### Unsure Respondents

As would be expected, respondents who were unsure (n=204) about using an app gave mixed reasons for and against app use. In response to the question of what would lead to a change of mind (or for them, decision-making), the most frequent response by far was the need for more information (27.5%). This was followed by a preference for a decentralized app (6.4%), being unsure what could change their mind (5.9%) and proof that the app is functional (5.4%). Some quotes from unsure participants are given here as illustrations of their viewpoints. As shown, respondents needed further information in a variety of areas including how the app would function, the data to be collected and its use, the risks to privacy, data security, and the impact on the phone battery, location tracking, and data usage.

#### More Information

Much more detailed understanding about how it works, and credibility of the organisation launching/running the app.

Info about how it works and why it matters.

More information on impact upon phone battery life and privacy (location tracking).

Very detailed, clear, public explanation of the App's findings.

More knowledge and a better understanding of the way it works.

Full disclosure about what is and isn't tracked and stored, and confidence in the people evaluating this and reporting it.

Complete honesty as to what happens to all the personal data collected.

I would happily use it if I understood more about what the potential risk to my personal information is and how that is mitigated.

#### Decentralized App

Decentralised data handling and storage.

Legally binding commitments on how data will be used and how long it will be stored. Fully anonymised decentralised system.

I'll use one once the tech has had a chance to bed in. I'd much prefer to use one which is coordinated with those in use in other countries so as to facilitate travel.

#### Being Unsure

Not sure but if there was some way to ensure the data would be safe (don’t trust gov to do as they say necessarily).

Not sure cos all authorities lie.

I'm not sure, a lot of reassurance that it's secure.

#### Proof it Works

Good evidence that it works and is safe.

Independent confirmation of adequate security and usefulness of the app.

If it was widely used and therefore accurate.

#### Other Reasons

A guarantee that the information would not be used for anything else and it was secured.

Assurance in law that my data would be solely used for contact tracing and that no private companies would have the right to hold or use my data.

Some very clear advice on how to install, use, etc. Support with what kind of phone is needed.

The price of a smart phone. It might be just easier for me to wear a mask and maintain physical distancing.

## Discussion

### What This Study Adds

To our knowledge, this is the only study on the use of a COVID-19 contact tracing app to use a mixed methods approach and combine qualitative and quantitative data collection and analysis at this scale. This is also the only known study focusing on Welsh citizens, thus adding Wales to the countries studied. In particular, allowing participants to provide open responses on their reasoning, and what would cause them to change their mind, demonstrated the value of asking more than closed questions and provided rich information at scale to augment the simple categorical answers. This information is important if policy and other decision-makers are to address and respond to concerns and to support use of a contact tracing app.

### Principal Findings

Our mixed methods survey among citizens of Wales found that over half (55.7%) of the respondents explicitly stated they planned to use a contact tracing app; a further 21% were unsure and just less than a quarter (23.3%) stated that they were unwilling. These values are based on actual responses plus inferred choices for those participants who did not answer the question. In contrast with the other closed questions in our survey, which were completed by over 95% of people, the response rate for this question was only 66%. This is interesting as it suggests some reluctance to respond to this question; nonetheless, almost all nonresponders gave reasons for being for, against, or unsure of using a contact tracing app in the free-text responses. The reasons for this are unknown, but it might indicate forms of response bias, such as acquiescence or social desirability, since after inferring from reasons, the proportions shifted toward unwillingness and unsureness. By comparison, almost three-fourth (74.8%) of participants in a multi-country survey using Likert scales stated they would probably or definitely download a contact tracing app [5]; over 67.5% of US citizens and 84% of Irish citizens indicated that they would probably or definitely download an app [6,7]. The US survey was also conducted in the United Kingdom, Germany, Italy, and France, with definite and probable intention to use rates at least as high as or higher than those in the United States [13].

In February 2021, the NHS Test and Trace program released the first detailed data about app use since it was rolled out in England and Wales in September 2020, and it reported that 21.7 million people had downloaded the app [14]. With the population of England and Wales being 59.4 million individuals [15], this indicates that 36.5% of the population downloaded the app. We refer to the combined figures for England and Wales because separate figures for Wales alone were not reported. The actual download figures are considerably lower than those found in our survey or in other surveys conducted in the United Kingdom [5,13]. It is widely recognized that a majority uptake is needed for optimum app efficacy; however, thus far, the figures fall far short. The reasons for this are not known, but they might be partly owing to varying representativeness, a tendency among respondents to provide the survey response seen as desirable, changes in viewpoints over time, and intention not being borne out by action for any reason.

In our survey, the top reasons in favor of app use were controlling spread of the virus, mitigating risks for others and for oneself, and increasing freedoms to enable society to open up. By comparison, the top reasons among some other surveys, which were based on predefined choices were as follows. In the US survey [6], protecting family and friends, knowing about the risk of infection, stopping the epidemic, and staying healthy were the top reasons. In the multi-country survey conducted by Altmann et al [5], the top reasons were protecting family and friends, stopping the epidemic, social responsibility, and knowing about the risk of infection. In the survey in Ireland [7], the top reasons were protecting family and friends, social responsibility, knowing about the risk of infection, and protecting oneself [7]. The top reasons for being against app use in our survey were mistrusting the government, concerns about data security and privacy, and doubts about app efficacy. Among other surveys, the top reasons were as follows. In the US survey conducted by Abeler et al [6]: concerns about government surveillance post pandemic, that no one else would use the app, phone being hacked, and increased anxiety. In the study by Altmann et al [5], the top 2 reasons were the same as those in the US survey, followed by increased anxiety and it being a major inconvenience to install the app. In O’Callaghan and colleagues’ study [7], the top reasons were surveillance by technology companies after the pandemic, a response that none of the options apply, government surveillance post pandemic, and the phone being hacked. Although category names differ, it can be seen that the top reasons for or against app use are similar across our and other surveys.

We included a question in our survey on what could lead people to change their mind on app use as we expected it to yield interesting results, given that this was a first-of-its-kind app and global context. As observed, 24% of people who downloaded the NHS app in England and Wales are not using it, which indicated a change of mind from being willing to being unwilling [16]. In the government data release showing 21.7 million people had downloaded the app, it was also revealed only about 16.5 million people (27.8%) were currently actively using it. It has been proposed that this discrepancy is due to a combination of people turning off the contact tracing capability, uninstalling the app or never actually activating it [14,16]; but the reasons for the change are not known. Of those in our survey willing to use an app, the top response for changing their mind from being willing to being unwilling was that nothing would cause them to do so, indicating their firm intentions. This was followed by assessing whether there was a breach in data security, if the app proved to be ineffective, and if their personal data were misused. These stated reasons may shed some light on the loss of app users as fear of breaches and misuse, as well as low uptake figures being seen as poor efficacy. The latter point is somewhat ironic and could create a self-fulfilling prophecy, one person at a time [9]. The same response (ie, that nothing would cause them to change their mind) was also the most frequent for those unwilling to change their mind to be willing. This was followed by the use of a decentralized app, being provided with suitable tech (ie, a smartphone, a network connection, or both), and data safety assurance. Since at the time our survey was conducted the intention was to introduce a centralized app in England and Wales, the subsequent change to and roll-out of a decentralized app might at least partly reduce unwillingness. However, the other difficulties and concerns remain to be addressed. Among the unsure, it was the need for more and clearer information, which can be seen as positive as it suggests it can be remedied through better communication. The actual reasons for the 24% loss in app users are not known, nor what proportion of people had previously been against using the app but had changed their mind to be in favor. Further studies are required to obtain information on these questions.

Since contact tracing apps require the use of a smartphone and a suitable network connection, digital inclusion and exclusion and underlying links with socioeconomic status, are important factors. In our survey, although 91.5% of overall respondents reported being smartphone users, this was only 78% among individuals aged 76-85 years and only 40% among those aged over 85 years. Not having, not wanting to, or being unable to buy, a smartphone, difficulties in using their smartphone, lack of knowledge on how to download and use an app, and lack of a reliable network connection were among the free-text reasons given for not using the app. At least one of these reasons was given by almost 10% (n=93) of our respondents. In April 2020, the Ada Lovelace Institute [17] published a rapid evidence review on the technical considerations and societal implications of using technology to transition from the COVID-19 crisis [18]. This included consideration of various issues relating to the use of contact tracing apps, among which were the potential exclusion of vulnerable groups and exacerbation of pre-existing health inequalities. The report highlighted that the effectiveness of digital contact tracing needed to be established, that effectiveness relies on a high level of accuracy and ubiquity, and is dependent on public trust and confidence. It further warned about societal and financial implications for individuals required to self-isolate and the possibility of fake contact warnings and other scams. The report concluded that there was (at the time of publication) insufficient evidence to support the use of digital contact tracing as an effective technology to support the pandemic response. It recommended clear government commitment to the following: privacy by design in app development and function, robust regulation and oversight, time limitation on contact tracing, purpose limitation in data use, clear guidance on the enforcement and use of digital contact tracing, and transparency to enable public scrutiny [18]. These concerns accord with many of those raised by our survey respondents. The data release on app use showed that 1.7 million people in England and Wales had been told to self-isolate as a result of using the NHS app, which health ministers estimate has prevented about 600,000 cases of the disease [16]. This is certainly good news, as is the change from a centralized to decentralized app model with regard to the preferences of our respondents. However, little is known about government achievements on other recommendations and on public involvement. Accepting that COVID-19 is having widespread and unequal serious impacts on individuals and societies, there are still ethical issues, such as the relationship between liberty and privacy to be addressed, and it has been shown that moral reasoning plays an important part in decision-making on app use [4,9]. Considering that government mistrust was the most frequent reason given by our respondents unwilling to use an app, and it was high among the reasons in other surveys, policy makers and other decision-makers need to increase efforts to engage with citizens, provide clearer information and act transparently if societies are to get the best from smartphone-based contact tracing apps. These issues will only become more important if added functionalities are introduced, such as vaccine status which is under discussion in the United Kingdom [19], in addition to the need for ongoing monitoring, since we have long progressed beyond common early thinking that the COVID-19 pandemic would end quickly.

### Limitations

As is common in one-time surveys, our work was based on a defined period and, as such, presents a snapshot of citizen views at that time. Further, the timing of the survey may have had an impact as it was early in the “first wave” of the pandemic and at a time when the NHSX model was the only model being tested at scale in the United Kingdom. As the data were collected anonymously, we cannot repeat the survey with the same respondents to compare their intentions with their actual decisions on the use of a contact tracing app. We acknowledge that our respondents are not fully representative of the people of Wales in terms of age profile, digital literacy, and ethnic heterogeneity. Other survey models were piloted with the aim of hearing from underrepresented groups, with some success, but the results are not reported here owing to adaptations in method reducing the viability of comparison with the HWW cohort.

### Recommendations

The following are some recommendations arising from our survey to inform decisions on enhancing the use of a contact tracing app to promote its effectiveness and build public trust. Although these arose from a survey with people in Wales, they are more widely applicable as in accordance with survey findings from other countries.

Concurrent with the transparency in a democratic society, there should be more engagement with the public to gain viewpoints, listen to concerns, and provide more information. This would also benefit decision-makers in developing transparent policy plans with social license.

There is an issue with digital inclusion among some groups, such as older people, being less likely to use a smartphone. In some cases, it is the lack of a smartphone or stable network connection, but for others it is a lack of knowledge on app use. For the latter, this could be at least partly addressed by an education program with straightforward information and a step-by-step guide to download and use the app.

The reasons people gave for being willing to use a contact tracing app demonstrate a keenness to help themselves, others, society, and the government to avoid the virus and control its spread. However, the reasons they might change their mind, notably, the need to safeguard against security breaches and data misuse, and to be able to demonstrate app effectiveness are critical to trust and success. Regularly updated reliable information is crucial to this.

The reasons people gave for their unwillingness to use an app were topped by mistrust in the government, followed by concerns about data security and privacy and the efficacy of the app. Policy and decision makers must address these issues and demonstrate trustworthiness if members of the public are to be confident their data are safe and that using an app is worthwhile.

In summary, we recommend greater public involvement in the development and implementation of policy and technologies from the outset and on an ongoing basis.

### Future Work

As a separate question alongside the survey, we asked respondents to indicate which topics interested them for an in-depth discussion and to email us outside the survey if they would like to take part. These topics were as follows: (1) what counts as acceptable use of digital technologies including apps, (2) the development and implementation of Wales-specific policy responses to COVID-19, (3) the potential benefits and challenges of using personal data gathered in the COVID-19 response for research purposes beyond the pandemic, (4) public engagement with proposed government strategies prior to implementation (and ongoing), (5) the impact of digital technologies introduced in response to the COVID-19 crisis on disadvantaged groups, and (6) the ethical challenges of designing, developing, and implementing technologies that support the exit strategy. The most frequently chosen was topic 3. Accordingly, we have embarked on deliberative public involvement [20] to ascertain public views on this topic to present to decision-makers in due course. The adapted survey formats (mentioned above) and their findings will be reported in a separate study.

### Conclusions

This is the only known citizen survey on the use of contact tracing apps to use a mixed methods approach, combining qualitative and quantitative data collection and allowing respondents to suggest their own reasons for and against app use, plus what would cause them to change their decision. Our findings show that citizens are intent on helping themselves, others, society, and government to avoid the virus and control its spread. The fact that contact tracing apps are necessarily smartphone-based raises issues of digital inclusion, such that participation is precluded for individuals who do not have a smartphone, have difficulty using one, or lack a stable network connection. However, the most prominent concerns raised about app use, namely, mistrusting the government, concerns about data security and privacy, and doubts about efficacy, could be addressed by greater efforts by policy and decision-makers to act openly, provide clearer information, and demonstrate trustworthiness. These actions are essential if the potential of contact tracing apps in contributing to controlling the pandemic are to be realized and may be useful in the ethical development and roll out of other health apps.

## Appendix

Multimedia Appendix 1 Survey questions.

## Abbreviations

HWW: HealthWise Wales
NHS: National Health Service
