# Supplementary material for: To Use or Not to Use a COVID-19 Contact Tracing App: Mixed Methods Survey in Wales
Source: JMIR Mhealth Uhealth. 2021 Nov 22;9(11):e29181. doi: 10.2196/29181 (PMC8610446; doi:10.2196/29181)
Supplement: Multimedia Appendix 1 [file mhealth_v9i11e29181_app1.docx]

**To use or not to use a COVID-19 contact tracing app: a mixed-methods survey in Wales**

**Appendix 1: Survey questions**

**Welcome**

It will take 2-4 minutes to complete this short survey

Please read the project summary and data protection statement at <https://www.healthwisewales.gov.wales/covid19/> before continuing.

**1. Consent**

I consent to the information provided in this survey being used solely for the research purposes and under the conditions set out in the project summary and accompanying data protection statement.

Yes: next question

No: Thank you message

**2. Gender**

Female

Male

Other- open text box to write preference or ‘prefer not to say’

**3. Age**

16-25

26-35

36-45

46-55

56-65

66-75

76-85

85+

**4. Ethnicity**

Asian or Asian British

Black, African, Black British or Caribbean

Mixed or multiple ethnic groups

White any

Other- open text box

**5. Location**

What is the first part of your residential postcode?

-Open text box (short)

**6. Are you considered to be high risk for COVID-19?**

(If self-assessed, or notified, high risk please answer yes)

Yes

No

**7. Have you had COVID-19 (either diagnosed or self-reported)?**

Yes

No

**8. Has someone you know had COVID-19 (either diagnosed or self-reported)?**

Yes

No

**9. Do you use a smartphone?**

Yes: Q11

No: Q10

**10. Would the introduction of app based COVID-19 contact tracing influence you getting a smartphone?**

Yes

No

**11. Are you aware of COVID-19 symptom tracking apps?**

(People use the app to report their health status and any symptoms of Covid19, which enables mapping of infections by geographical area)

Yes

No

**12. Do you currently use a symptom tracking app?**

For example the Covid Symptom Study (formerly Covid Symptom Tracker) also known as ZOE.

Yes

No

**13. Are you aware of plans to introduce a contact tracing app in the UK?**

(Contact tracing apps are designed to trace who a person with COVID-19 symptoms has been in contact with) For example NHSX app

Yes

No

**14. Do you feel you currently have sufficient knowledge of the potential benefits and risks, to decide whether or not to use a contact tracing app?**

Yes

No

**15. Do you plan to use a contact tracing app when made available?**

Yes

No

Unsure

**16. Please provide up to three main reasons for your decision to use/not use a contact tracing app**

Open text box

**17. What, if anything, would change your decision regarding using a contact tracing app?**

Open text box
